# Effect of Egg-Coating Material Properties by Blending Cassava Starch with Methyl Celluloses and Waxes on Egg Quality

**DOI:** 10.3390/polym13213787

**Published:** 2021-11-01

**Authors:** Pornchai Rachtanapun, Nattagarn Homsaard, Araya Kodsangma, Noppol Leksawasdi, Yuthana Phimolsiripol, Suphat Phongthai, Julaluk Khemacheewakul, Phisit Seesuriyachan, Thanongsak Chaiyaso, Suwit Chotinan, Pensak Jantrawut, Warintorn Ruksiriwanich, Sutee Wangtueai, Sarana Rose Sommano, Wirongrong Tongdeesoontorn, Kittisak Jantanasakulwong

**Affiliations:** 1 School of Agro-Industry, Faculty of Agro-Industry, Chiang Mai University, Mae-Hea, Mueang, Chiang Mai 50100, Thailand; Pornchai.r@cmu.ac.th (P.R.); buzz_buss_@hotmail.com (N.H.); mymild_araya@yahoo.com (A.K.); noppol@hotmail.com (N.L.); yuthana.p@cmu.ac.th (Y.P.); suphat.phongthai@cmu.ac.th (S.P.); Looktarn005@hotmail.com (J.K.); phisit.s@cmu.ac.th (P.S.); thachaiyaso@hotmail.com (T.C.); 2 Center of Excellence in Materials Science and Technology, Faculty of Science, Chiang Mai University, Chiang Mai 50200, Thailand; 3 Cluster of Agro Bio-Circular-Green Industry (Agro BCG), Chiang Mai University, Chiang Mai 50100, Thailand; suwit.c@cmu.ac.th (S.C.); pensak.amuamu@gmail.com (P.J.); warintorn.ruksiri@cmu.ac.th (W.R.); sutee.w@cmu.ac.th (S.W.); sarana.s@cmu.ac.th (S.R.S.); 4 Faculty of Veterinary Medicine, Chiang Mai University, Chiang Mai 50200, Thailand; 5 Department of Pharmaceutical Sciences, Faculty of Pharmacy, Chiang Mai University, Chiang Mai 50200, Thailand; 6 College of Maritime Studies and Management, Chiang Mai University, Samut Sakhon 74000, Thailand; 7 Plant Bioactive Compound Laboratory (BAC), Department of Plant and Soil Sciences, Faculty of Agriculture, Chiang Mai University, Chiang Mai 50200, Thailand; 8 School of Agro-Industry, Mae Fah Luang University, Chiang Rai 57100, Thailand; wirongrong.ton@mfu.ac.th

**Keywords:** *Manihot esculenta*, carboxymethyl cellulose, paraffin, Haugh unit, weight loss, freshness, edible film

## Abstract

An egg-coating material was developed to extend the shelf-life and freshness of eggs by blending cassava starch (CS) with gelling agents and waxes. The effects of the properties of this egg coating on egg quality were investigated. Hydroxypropyl methylcellulose (HPMC), carboxymethyl cellulose (CMC), beeswax, and paraffin wax were used. CS blended with low-molecular-weight paraffin (Paraffin(L)) and CMC coating material displayed a tensile strength of 4 MPa, 34% elongation at break, 0.0039 g day^−1^ m^−2^ water vapor permeability, and a water contact angle of 89° at 3 min. Eggs coated with CS/CMC/Paraffin(L) solutions had a Haugh unit value of 72 (AA grade) and exhibited a weight loss of 2.4% in 4 weeks. CMC improved the compatibility of CS and Paraffin(L). This improvement and the hydrophobicity of Paraffin(L) provided suitable mechanical and water-resistance properties to the coating material that helped to maintain the quality of the coated AA-grade eggs with low weight loss for 4 weeks.

## 1. Introduction

Eggs are one of the best natural sources of high-quality proteins, vitamins, antioxidants, carotenoids, and phospholipids [1]. Of all eggs, chicken eggs are most widely consumed by humans because they are a reasonable source of high-quality protein and contain diverse nutrients [2]. Aging of an egg begins once the egg is laid, characterized by changes in functional properties. The effect of an egg-coating material on the eggshell depends on temperature and storage time [3]. The coating biomaterials used in the egg-production industry may be lipid-, protein-, or polysaccharide-based. Biomaterials with properties amenable to egg-coating include starch [4,5,6], carboxymethyl cellulose (CMC) [7,8,9], carboxymethyl chitosan [10], carboxymethyl bacterial cellulose [11], sericin [12,13], keratin [14], fibroin [15], and pectin [16,17,18,19].

Polysaccharide-based coatings are commonly used due to the fact of their high flexibility, thinness, and high transparency. Polysaccharides have been synthesized from microbes [20], lactic acid bacteria [21], and purple glutinous rice bran [22]. A study investigated the ability of numerous coating materials, such as mineral oil, waxes, shellac, and chitosan, to cover the pores of eggshells and maintain egg quality [23]. Many natural products are incorporated into coating materials for eggshells to prolong the shelf life of eggs. These materials include proteins [24], protein isolates [25], propolis [26,27], whey protein and zein [28,29,30], CMC [31], and chitosan [32,33]. Coating with oil, immersion in liquids, refrigeration, and dry packing are other examples of methods used to maintain egg quality. Temperature control and storage under refrigerated conditions are effective methods to preserve egg quality [34]. The most common forms of lipid used in edible coatings are waxes [35].

Albumin height and egg weight can be used to calculate the Haugh unit (HU) value, which indicates albumen quality [36]. An HU value of approximately 80 indicates a fresh egg of high quality. The HU is influenced by storage time [37]. Egg quality depends on weight loss due to the fact of moisture penetration. When egg weight is high, profitability is also high, owing to reduced water loss [38,39,40,41].

Polymer-solution blending is an effective method for preparing egg-coating materials. Biopolymer materials are blended with a gelling agent and a water-resistant polymer. Some edible biopolymers have been used to prepare egg coatings including hydroxypropyl methylcellulose (HPMC) [42], cassava starch (CS) [31], and chitosan [33]. HPMC has been used as a gelling agent [43]. Starch blended with CMC or HPMC improves the mechanical properties of the blends [44,45]. The water resistance of egg-coating materials is improved using mineral oil [32], soybean oil [33], fatty acids [42], and shellac [23]. Waxes are an edible natural biopolymer with hydrophobic properties [46] and are a suitable biomaterial to improve the water resistance of coating materials. Waxes exist in a solid state at ambient temperature and transform to a liquid state at higher temperatures, while low-molecular-weight (Mw) waxes are in a liquid state at room temperature. Important considerations while preparing egg-coating material for prolonging egg shelf-life include safety, mechanical properties, water resistance, migration, and production cost of the egg-coating material. Many formulations with different properties have been tested: an edible CS/CMC/fatty acid blend used as an egg-coating material has been reported [31]. Rice protein and Brazilian green propolis were used to prepare an additional egg-coating material that delayed the loss of egg quality by preventing moisture loss [47]. A chitosan/beeswax/essential oil blend prevented bacterial growth and extended the shelf-life of eggs [48]. Starch with an added agent that prevents microbial growth has also been reported [49]. Furthermore, pulsed-light technology has been used to prevent bacterial contamination and extend egg shelf-life [50]. However, blending waxes with starch and methyl cellulose to prepare egg-coating materials has not been reported before.

In this study, CS was blended with gelling agents and waxes to prepare new edible egg-coating materials. This coating material was designed to be environmentally friendly, safe, and highly water-resistant with good mechanical properties and low production costs. CS was selected as the main matrix carbohydrate polymer for the coating formulation because of its high purity, low cost, chemical modification ability, and abundance. HPMC and CMC were incorporated as gelling agents and emulsifiers for the egg coating, and glycerol was added as a plasticizer. Beeswax and paraffin waxes were used to improve the water resistance of the coating formulation. Low-Mw paraffin wax (Paraffin(L)) was used to compare the chain-length effect of paraffin. The water vapor permeability (WVP), water droplet contact angle, and tensile properties of the egg-coating film were observed. The egg quality was examined based on HU values and weight loss after 4 weeks of storage at 25 °C. Moreover, the relationship of material properties to egg quality was evaluated. 

## 2. Materials and Methods

### 2.1. Materials and Preparation of Coating Solutions

Fresh eggs from 20-week old hens were obtained on the day of laying from R.P.M. Farm & Feed Co., Ltd. (Hang Dong, Chiang Mai, Thailand). CS (Dragon Fish brand) with a moisture content of 11% total weight, amylose/amylopectin content of 17%/83%, and a Mw of 1.34 × 10^8^ g/mol was obtained from Tong Chan Registered Ordinary Partnership (Bangkok, Thailand). Glycerol (99%) and beeswax were purchased from Union Science Co., Ltd. (Chiang Mai, Thailand). CMC (grade 700, degree of substitution (DS) 0.8, and Mw 270,000 g/mol) was obtained from CP Kelco Co., Ltd. (Äänekoski, Finland). HPMC (Methocel, E4M Premium, DS/MS = 1.9/0.23, and Mw = 351,490 g/mol) was obtained from Dow Chemical Co., Ltd. (Midland, MI, USA). Paraffin and Paraffin(L) were purchased from Hong Huat Co., Ltd. (Bangkok, Thailand).

### 2.2. Coating and Film Preparation

Coating materials and films were formulated as outlined in Table 1. Coating materials were mixed in the following order of priority: water, CS, glycerol, gelling agents, and waxes. Each mixture was homogenized in 80 °C distilled water at 500 rpm for 20 min using an overhead stirrer. Each prepared coating solution was used to coat the eggs with the wiping method described below, and the eggs were then placed on racks to dry at 28 °C. Eggs were selected from 20 week old hens. When eggs arrived on the first day from the farm, they were screened for surface cleanliness, cracks, and breakage. Before coating, egg weight (range: 66–70 g) was recorded. During the wiping procedure for coating, eggs were dipped into a coating material that was then wiped onto the eggshells. Once dry, the eggs were stored at 25 ± 3 °C and a relative humidity (RH) of 65 ± 2%. For each formulation, five eggs were examined weekly over 4 weeks of storage. Uncoated eggs were also stored and observed under the same conditions and for the same durations. Coated and uncoated eggs were periodically analyzed for weight loss and albumen quality. The coating material films were prepared to measure WVP, contact angle, and tensile properties. A film-forming solution was cast by the solvent-casting method [51], followed by film drying at 60 °C for 24 h.

### 2.3. Tensile Properties of Films

Tensile properties were determined following the JISK-6251-7 standard using an Instron Universal Testing System (H1KS; Surrey, UK) at a crosshead speed of 2 mm min^−1^ with bone-shaped samples. The sample film was prepared by solution-casting and cut using a mold. The width, thickness, and gauge length were 2 mm, 0.2 mm, and 10 mm, respectively. The experiments were performed in quintuplicate. Film specimens were conditioned at 30 ± 1 °C and 50 ± 2% RH for 24 h.

### 2.4. Measurement of Haugh Unit

Broken-out egg measurements were performed using a Haugh gauge. HU values of the coated and uncoated eggs were measured initially and at specific sampling periods. Five samples of coated and uncoated treatments were observed at each storage interval. HU was calculated using Equation (1):

(1) $$\mathrm{HU}=100\times \log(h-1.7w^{0.37}+7.6)$$

where H is the albumen height (mm), and w is the weight of the egg (g) [52].

### 2.5. Determination of Weight Loss

Eggs were weighed immediately before storage and at specific storage intervals. The same egg was weighed at each storage time, and the weight loss percentage was calculated. Five replicates were observed for each condition. From these data, the average weight loss percentage of coated and uncoated eggs was calculated as the difference between initial (*W_i_*) and final (*W_f_*) egg weights divided by *W_i_* and multiplied by 100 (Equation (2)) [31].

(2) $$\mathrm{Weight}\;\mathrm{loss}\;\left(\%\right)=\frac{W_{i}-W_{f}\;\;}{W_{i}}\times 100$$

### 2.6. Measurement of Contact Angle

The water contact angle was measured using drop-shape analysis with a model DSA30E device (Krüss Co., Ltd., Hamburg, Germany). Samples were conditioned at 50 ± 2% RH at 30 ± 1 °C for 24 h. Images of the water contact angle between the water droplet and film surface were obtained automatically every 10 s for 1 min. Five replicates for each sample were observed.

### 2.7. Water Vapor Transmission Rate (WVTR) and Water Vapor Permeability (WVP)

The *WVTR* was determined according to the ASTM E96 protocol for water vapor transmission of materials using the gravimetric method. Silica gel (10 g) was placed in a cup, covered with a specimen (a circle of film sample with a diameter of 60 mm), and sealed with paraffin wax. The cup samples were kept in a desiccator at 65 ± 2% RH and 25 ± 3 °C. The weight of the cup samples was measured, and samples were reweighed daily for 5 days (ASTM E104). A constant RH was achieved using a saturated sodium chloride solution. The slope of the linear relationship between the dependent variable of weight gain and the independent variable of time was obtained from the data and used to calculate the *WVTR* using Equation (3) [53]:

(3) $$WVTR=\frac{x}{\left(t\times A\right)}$$

where *WVTR* is expressed as g day^−1^ m^−2^. The term *x*/*t* was measured by linear regression analysis from the points of time and weight gain over a constant rate period, with *A* as the area of the exposed film. Five replicates for each sample were observed. *WVP* was measured using Equation (4) [53]:

(4) $$WVP=\frac{\left(WVTR\times l\right)}{\Delta P}$$

where *WVP* is expressed as g day^−1^ m^−1^ Pa^−1^, *l* is the film thickness, and Δ*P* is the water vapor pressure differential across the film at 25 °C.

### 2.8. Statistical Analysis

All results were analyzed by one-way analysis of variance using SPSS software (IBM, Armonk, NY, USA). Statistically significant differences (*p* < 0.05) were evaluated using Duncan’s test. Five replicates for each sample were used for the evaluations.

## 3. Results and Discussion

### 3.1. Tensile Properties of Films

**Figure 1:**
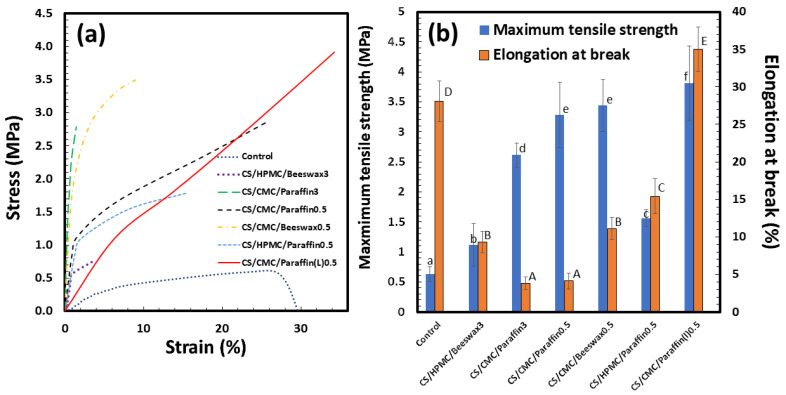
Tensile properties of coating materials: (**a**) Stress–strain curve and (**b**) maximum tensile strength and elongation at break (*n* = 5). The control indicates the starch/glycerol blend. Means with different lowercase letters of elongation at break and the uppercase letters of maximum tensile strength were significantly different (*p* < 0.05).

The tensile properties of the coating materials were examined to determine suitable physical properties of coating films for use as egg-coating agents. Figure 1 shows the stress–strain curves of the control (i.e., CS/glycerol), CS/HPMC/Beeswax3, CS/CMC/Paraffin3, CS/CMC/Paraffin0.5, CS/CMC/Beeswax0.5, CS/HPMC/Paraffin0.5, and CS/CMC/Paraffin(L)0.5 films. CS/HPMC/Beeswax3 exhibited a low tensile strength (0.7 MPa) and elongation at break (4%), whereas CS/CMC/Paraffin3 exhibited a tensile strength of 2.8 MPa and elongation at break of 4% with the highest modulus (slope at the first stage of the stress–strain curve). Poor compatibility between HPMC and beeswax was indicated in the CS/HPMC/Beeswax3 sample, whereas the high stiffness of CMC and paraffin increased the modulus of the CS/CMC/Paraffin3 sample [54]. CS/CMC/Beeswax0.5 showed a high modulus with a tensile strength at a break of 3.5 MPa. Mixing Paraffin0.5 with the CS/HPMC and CS/CMC samples increased the elongation at break of the coating materials. CS/CMC/Paraffin(L)0.5 displayed the lowest modulus and highest tensile strength (4.0 MPa) and elongation at break (34%), indicating good suitability of the properties of the material for coating film preparation for the eggshell surface. These results suggest that Paraffin(L) increased the flexibility of the CS/CMC blend as a plasticizer. The strong compatibility between CMC and Paraffin(L) can be attributed to the formation of interactions between the –COOH and –COO^−^Na^+^ groups of CMC with the –OH groups of Paraffin(L). Interactions between the –COO^−^ groups of CMC and –OH groups have been reported separately [45,55].

### 3.2. Haugh Unit

**Figure 2:**
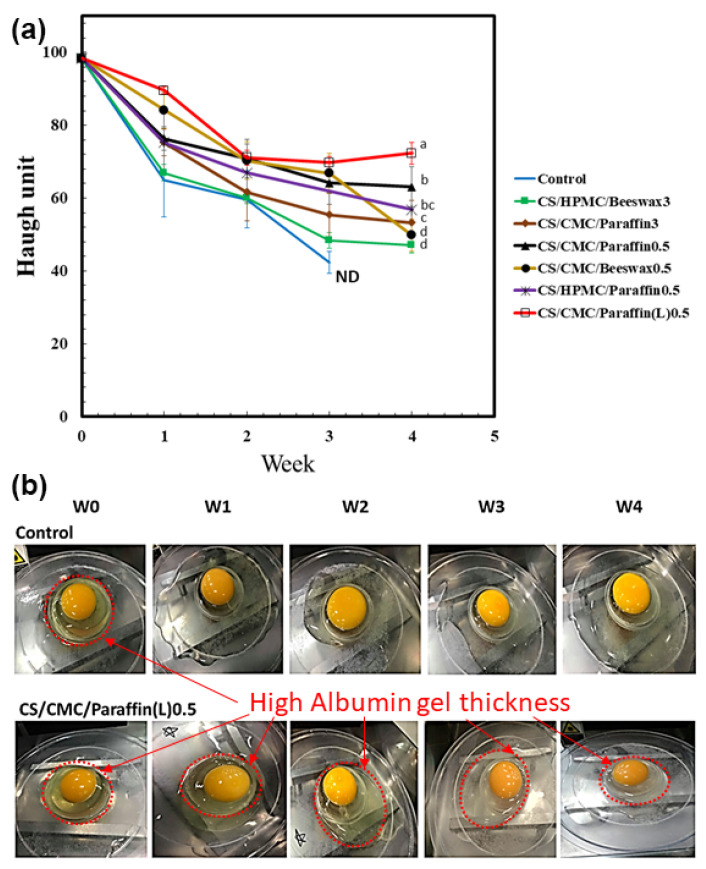
Quality of the coated and uncoated eggs: (**a**) Variation in Haugh units (HUs) (*n* = 5) of cassava starch (CS) blended with hydroxypropyl methylcellulose (HPMC), carboxymethyl cellulose (CMC), beeswax, paraffin, and low-molecular-weight paraffin wax (Paraffin(L)) during storage from day 0 to week 4 at 25 ± 3 °C and 65 ± 2% relative humidity. The control indicates uncoated eggs. ND indicates not detected. Means with different lowercase superscript letters were significantly different (*p* < 0.05). (**b**) Images of uncoated eggs (control) and eggs coated with CS/CMC/Paraffin(L)0.5 from day 0 to week 4.

The HU values of eggs coated with the CS formulations during 4 weeks of storage at 25 ± 3 °C are shown in Figure 2a. The grade of the egg was determined based on the HU values as follows: AA > 72, A = 72–60, B = 59–31, and C ≤ 30 [56]. The HU values of eggs coated with CS/HPMC/Beeswax3 decreased rapidly from 98 (grade AA) to 67 (grade A) in the first week and remained at grade B (42) until week 4. The CS/HPMC/Paraffin0.5 sample displayed decreased HU values from 98 to 56 by week 4. The CS/HPMC/Paraffin0.5 sample demonstrated higher HU values than those of the CS/HPMC/Beeswax3 sample in week 1; however, the values gradually decreased during the storage period. In week 4, the HU values according to sample coating were: CS/CMC/Paraffin3, 53 (grade B); CS/CMC/Beeswax0.5, 49 (grade B); CS/CMC/Paraffin0.5, 64 (grade A); CS/CMC/Paraffin(L)0.5, 72 (grade AA). Images of uncoated eggs and eggs coated with CS/CMC/Paraffin (L)0.5 are shown in Figure 2b. The thickness of the albumin gel was pronounced with the CS/CMC/Paraffin(L)0.5 coating. The albumin thickness was minimal in uncoated samples throughout the 4 week storage period. The stability of the HU values of coated eggs during 4 weeks of storage has been reported for A grade at 25 °C [25,31,42] and for AA-grade eggs at 20 °C [24]. In the present study, the CS/CMC/Paraffin(L) coating extended the preservation of egg quality at grade AA for 4 weeks due to the hydrophobicity of Paraffin(L) and strong compatibility between Paraffin(L) and CMC via interaction of the two materials. Paraffin(L) acted as a plasticizer for the CS/CMC coating film, providing it with the flexibility to fill and cover the eggshell’s pores. The high hydrophobicity of Paraffin(L), strong compatibility of CMC and Paraffin(L), eggshell pore-filling, and the excellent physical properties of the CS/CMC/Paraffin(L)0.5 coating maintained the HU value and egg quality.

### 3.3. Weight Loss

**Figure 3:**
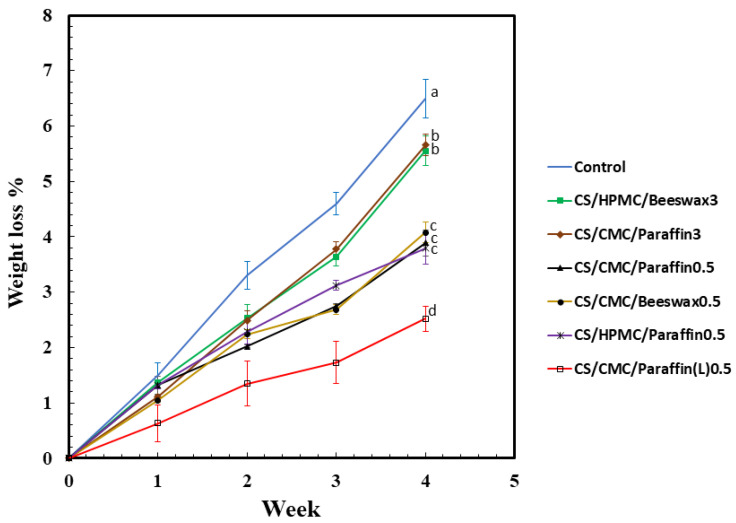
Variation in weight loss (%; *n* = 5) of eggs coated with cassava starch (CS) blended with hydroxypropyl methylcellulose (HPMC), carboxymethyl cellulose (CMC), beeswax, paraffin, and low-molecular-weight paraffin wax (Paraffin(L)) during 4 weeks of storage at 25 ± 3 °C and 65 ± 2% relative humidity. The control indicates uncoated eggs. Means with different lowercase superscript letters were significantly different (*p* < 0.05).

Egg weight is an important factor in determining egg quality. The weight loss of eggs coated with CS blended with HPMC, CMC, beeswax, paraffin, and Paraffin(L) over 4 weeks of storage at 25 °C is summarized in Figure 3. Weight loss increased during the retention period for all samples. Uncoated eggs showed 6.5% weight loss due to the loss of moisture via the eggshell’s pores. Weight losses in the eggs coated with the CS/HPMC/Beeswax3 and CS/CMC/Paraffin3 samples were 5.5% and 5.8%, respectively, after 4 weeks of storage. CS/CMC/Paraffin0.5, CS/CMC/Beeswax0.5, and CS/HPMC/Paraffin0.5 exhibited weight losses of 3.8%, 4.0%, and 3.7%, respectively. The CS/CMC/Paraffin(L) sample maintained a low egg weight loss (2.4%) throughout 4 weeks of storage. The CS/HPMC/Beeswax3 and CS/CMC/Paraffin3 groups showed a high weight loss at 4 weeks of storage due to the high number and larger sizes of wax particles relative to eggshell pore sizes. Paraffin and beeswax samples of 0.5% showed lower weight loss at 4 weeks of storage than 3% samples owing to hole-filling of the eggshell’s pores with the smaller size of wax. HPMC- and CMC-coated eggs did not significantly differ in terms of weight loss. The compatibility between CS and Paraffin(L) was shown to improve when CMC was used as an emulsifier. The low molecular weight of Paraffin(L) showed high compatibility with the CS/CMC blend and improved water-barrier activity due to the small molecule size and hydrophobicity of Paraffin(L), respectively. The combination of CS blended with CMC and Paraffin(L) prevented egg weight loss by filling eggshell pores with the highly water-resistant coating material, which lowered the evaporation rate of the water inside the eggs. The prevention of egg weight loss using highly water-resistant coating materials has also been previously reported [25,31,57].

### 3.4. Contact Angle Measurements

**Figure 4:**
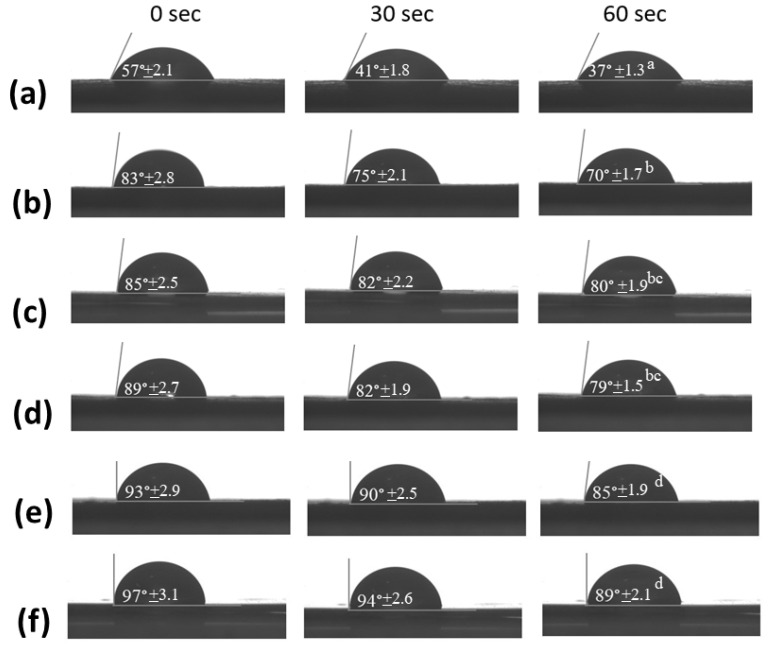
Water contact angles (*n* = 5) of (**a**) CS/HPMC/Beeswax3, (**b**) CS/CMC/Paraffin3, (**c**) CS/CMC/Paraffin0.5, (**d**) CS/CMC/Beeswax0.5, (**e**) CS/HPMC/Paraffin0.5, and (**f**) CS/CMC/Paraffin(L)0.5. Means with different lowercase superscript letters were significantly different (*p* < 0.05).

The contact angle was selected to observe the surface tension of the coating material films. Surface tension can prevent the penetration of moisture through the eggshell. Characteristic images of the water-droplet profiles of the egg-coating materials are shown in Figure 4. The CS/HPMC/Beeswax3-coated samples displayed a water contact angle of 57°. The angle decreased rapidly to 41° at 30 s and 37° at 60 s (Figure 4a). The findings indicate the incompatibility between HPMC and beeswax, which is related to the tensile properties of the CS/HPMC/Beeswax3 film (Figure 1). The CS/CMC/Beeswax0.5-coated samples displayed a water droplet contact angle of 89°, which decreased to 82° at 30 s and 79° at 60 s (Figure 4d). The CS/HPMC/Paraffin0.5 coating presented a contact angle of up to 93°, which gradually decreased to 90° at 30 s and 85° at 60 s (Figure 4e). CS/HPMC and CS/CMC blended with paraffin displayed higher surface tensions than did samples blended with beeswax due to the strong compatibility of paraffin and methyl celluloses. The CS/CMC/Paraffin(L)0.5 film displayed the highest contact angle (97°), which gradually decreased to 94° at 30 s and 89° at 60 s (Figure 4f). The high water droplet contact angle of the CS/CMC/Paraffin(L)0.5 coating was due to the hydrophobicity of Paraffin(L) and the strong compatibility of CMC and Paraffin(L). CMC improved the surface tension and water resistance of CS/Paraffin(L) due to the combination of polar and non-polar groups in CMC used as an emulsifier. The high water resistance of the CS/CMC/Paraffin(L)0.5 coating improved the HU value and quality of the eggs. The high water resistance of the coating materials that helped to maintain the HU value and quality of the eggs has also been reported elsewhere [28].

### 3.5. WVTR and WVP of Coating Films

**Figure 5:**
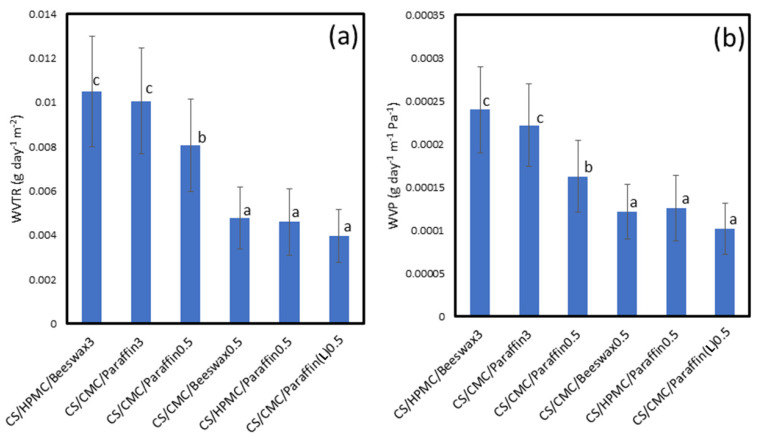
Water-resistance properties of coating materials: (**a**) water vapor transmission rate (*WVTR*) and (**b**) water vapor permeability (*WVP*) (*n* = 5). Means with different lowercase superscript letters were significantly different (*p* < 0.05).

The *WVTR* of a coating film is an important factor related to the hydrophilicity or hydrophobicity of the material. Egg quality can be controlled by moisture exchanged through the eggshell during storage. A hydrophilic film has a higher *WVTR* (lower water vapor barrier) than that of a hydrophobic film [58,59,60]. The *WVTR* values of the various coated films examined are shown in Figure 5a. The CS/HPMC/Beeswax3 film displayed the highest *WVTR* (0.01050 g day^−1^ m^−2^). This was due to the fact of phase separation resulting from the difference in polarity between HPMC and beeswax. Blending CS with HPMC and paraffin decreased the *WVTR* of the coating material due to the stronger compatibility of HPMC with paraffin than that of HPMC with beeswax. However, excessive amounts of paraffin wax did not improve the water resistance of the coating film due to the increase in the free volume inside the films. The CS/CMC/Beeswax0.5 film displayed a lower *WVTR* (0.00477 g day^−1^ m^−2^) and *WVP* (0.00012 g day^−1^ m^−1^ Pa^−1^) (Figure 5b) than those of the CS/CMC/Paraffin blends due to the high hydrophobicity of beeswax and compatibility of this material with CMC. CS/CMC/Beeswax0.5, CS/HPMC/Paraffin0.5, and CS/CMC/Paraffin(L)0.5 displayed low values of *WVTR* and *WVP*. This reflects the strong compatibility of methyl celluloses and waxes, the hydrophobicity of waxes, and the small free volume inside the films [61]. Only the CS/CMC/Paraffin(L)0.5 coating helped to maintain the HU value at grade AA for 4 weeks, because Paraffin(L) improved the compatibility, mechanical properties, flexibility, surface tension, and water resistance of the film. Our collective findings demonstrate that coating eggs with CS/CMC/Paraffin(L)0.5 helps to cover the eggshell’s pores, prevent moisture and weight loss, and maintain egg quality at grade AA for 4 weeks when stored at 25 ± 3 °C and 65 ± 2% RH.

## 4. Conclusions

An egg-coating material was successfully developed using a CS, CMC, and Paraffin(L) blend. The new edible coating material extended the shelf-life and quality of grade AA eggs for 4 weeks at 25 °C. CMC improved the compatibility between CS and Paraffin(L) via interaction between the –COOH and –COO^−^Na^+^ groups of CMC with the –OH groups of Paraffin(L), which improved the mechanical properties of the coating material. Small molecules of Paraffin(L) provided flexibility to coating material, acting as a plasticizer. Egg-coating materials using Paraffin(L) had advantages, such as flexibility and water resistance, which facilitated full coverage of eggshell pores, maintenance of the HU value, and reduced the weight loss of the eggs. The high compatibility of CS/CMC/Paraffin(L) and the hydrophobicity of Paraffin(L) increased the surface tension and reduced the water permeability of the coating material. CMC improved the compatibility of the CS/CMC/Paraffin(L) coating material, while Paraffin(L) acted as a plasticizer and provided waterproofing. High compatibility, tensile properties, flexibility, and the water resistance of the coating material prevented a reduction in the quality of the coated eggs. This coating material can therefore be applied in the egg-production and egg-distribution industries.

## Figures and Tables

**Table 1:** Composition of the coating materials made from cassava starch (CS), glycerol, carboxymethyl cellulose (CMC), hydroxypropyl methylcellulose (HPMC), beeswax, and paraffin wax.

Formulation	Composition (w/v%)					
	CS	Glycerol	CMC	HPMC	Beeswax	Paraffin
CS/HPMC/Beeswax3	6	2	-	1	3	-
CS/CMC/Paraffin3	6	2	1	-	-	3
CS/CMC/Paraffin0.5	6	2	1	-	-	0.5
CS/CMC/Beeswax0.5	6	2	1	-	0.5	-
CS/HPMC/Paraffin0.5	6	2	-	1	-	0.5
CS/CMC/Paraffin(L)0.5	6	2	1	-	-	0.5

## Data Availability

The data presented in this study are available on request from the corresponding author.

## Abbreviations

CMC = carboxymethyl cellulose; CS = cassava starch; HPMC = hydroxypropyl methyl cellulose; HU = Haugh unit; Paraffin(L) = low-molecular-weight paraffin; WVP = water vapor permeability; WVTR = water vapor transmission rate.
